# Potential Circumstances Associated With Moral Injury and Moral Distress in Healthcare Workers and Public Safety Personnel Across the Globe During COVID-19: A Scoping Review

**DOI:** 10.3389/fpsyt.2022.863232

**Published:** 2022-06-13

**Authors:** Yuanxin Xue, Jillian Lopes, Kimberly Ritchie, Andrea M. D’Alessandro, Laura Banfield, Randi E. McCabe, Alexandra Heber, Ruth A. Lanius, Margaret C. McKinnon

**Affiliations:** ^1^Temerty Faculty of Medicine, University of Toronto, Toronto, ON, Canada; ^2^Faculty of Health Science, McMaster University, Hamilton, ON, Canada; ^3^Psychology, Neuroscience and Behaviour Graduate Program, McMaster University, Hamilton, ON, Canada; ^4^Department of Psychiatry and Behavioural Neurosciences, McMaster University, Hamilton, ON, Canada; ^5^Homewood Research Institute, Guelph, ON, Canada; ^6^Neuroscience Graduate Program, McMaster University, Hamilton, ON, Canada; ^7^Health Sciences Library, McMaster University, Hamilton, ON, Canada; ^8^St. Joseph’s Healthcare Hamilton, Hamilton, ON, Canada; ^9^Veterans Affairs Canada, Ottawa, ON, Canada; ^10^Department of Psychiatry, University of Ottawa, Ottawa, ON, Canada; ^11^Department of Psychiatry, Western University of Canada, London, ON, Canada; ^12^Lawson Health Research Institute, London, ON, Canada

**Keywords:** COVID-19, healthcare workers, public safety personnel, moral injury, moral distress, scoping review, global

## Abstract

Healthcare workers (HCWs) and public safety personnel (PSP) across the globe have continued to face ethically and morally challenging situations during the COVID-19 pandemic that increase their risk for the development of moral distress (MD) and moral injury (MI). To date, however, the global circumstances that confer risk for MD and MI in these cohorts have not been systematically explored, nor have the unique circumstances that may exist across countries been explored. Here, we sought to identify and compare, across the globe, potentially morally injurious or distressful events (PMIDEs) in HCWs and PSP during the COVID-19 pandemic. A scoping review was conducted to identify and synthesize global knowledge on PMIDEs in HCWs and select PSP. Six databases were searched, including MEDLINE, EMBASE, Web of Science, PsychInfo, CINAHL, and Global Health. A total of 1,412 articles were retrieved, of which 57 articles were included in this review. These articles collectively described the experiences of samples from 19 different countries, which were comprised almost exclusively of HCWs. Given the lack of PSP data, the following results should not be generalized to PSP populations without further research. Using qualitative content analysis, six themes describing circumstances associated with PMIDEs were identified: (1) Risk of contracting or transmitting COVID-19; (2) Inability to work on the frontlines; (3) Provision of suboptimal care; (4) Care prioritization and resource allocation; (5) Perceived lack of support and unfair treatment by their organization; and (6) Stigma, discrimination, and abuse. HCWs described a range of emotions related to these PMIDEs, including anxiety, fear, guilt, shame, burnout, anger, and helplessness. Most PMIDE themes appeared to be shared globally, particularly the ‘Risk of contracting or transmitting COVID-19’ and the ‘Perceived lack of support and unfair treatment by their organization.’ Articles included within the theme of ‘Stigma, discrimination, and abuse’ represented the smallest global distribution of all PMIDE themes. Overall, the present review provides insight into PMIDEs encountered by HCWs across the globe during COVID-19. Further research is required to differentiate the experience of PSP from HCWs, and to explore the impact of social and cultural factors on the experience of MD and MI.

## Introduction

The COVID-19 pandemic has brought about unprecedented challenges for all citizens globally, with healthcare workers (HCWs; including nurses, physicians, personal support workers, social workers, etc.) (1) and public safety personnel (PSP; including police and correctional officers, firefighters, paramedics, etc.) (2) at the forefront of efforts to manage, contain, and remediate healthcare and societal impacts. HCWs and PSP have encountered ethically and morally challenging situations related to the unique circumstances of the COVID-19 pandemic. For example, global shortages of personal protective equipment (PPE) (3) forced HCWs and PSP to balance personal safety with their duty to the public (4–7). Similarly, perceived uneven and inequitable distribution of care in the face of shortages of life-saving resources has appeared particularly distressing, carrying heavy moral weight for HCWs (4, 5, 8, 9). Novel and challenging interactions with distressed families, patients, and the public that may include issues surrounding dying alone and enforcing limited visitation policies, have also been commonly described (10, 11). Given increased exposure to potentially traumatic and morally challenging events during the pandemic (12, 13), beyond that anticipated in these professions, it appears that the risk of developing COVID-19 related moral distress (MD) and/or moral injury (MI) may be exacerbated among HCWs and PSP. Despite this increased exposure and risk, scant literature exists to identify potentially morally injurious/distressful events (PMIDEs), shared across the globe. Moreover, unique PMIDEs across countries or continents remain to be identified. The present scoping review aimed to address these gaps in the literature.

The concept of MD originated in healthcare literature, being first described as the psychological distress that arises in a situation where one is constrained from pursuing the right course of action (14). Since then, a variety of definitions of MD have been proposed, spanning numerous professions. Morley et al. (15) surmised MD as arising from the experience of a “moral event” (e.g., a moral dilemma or moral uncertainty) which has a direct causal relationship with an experience of “psychological distress.” MD is associated with a range of negative sequelae, including lower job satisfaction, greater intention to leave one’s profession, reduced psychological empowerment and autonomy, and negative feelings that include anger, guilt, and powerlessness (16–18).

The concept of MI, which originated in the military context (19), has also assumed various definitions in recent decades, evolving independently from MD. Working from a syndromal perspective, Jinkerson (20) defined MI as the psychological, behavioral, interpersonal, and existential issues that arise following perceived violations of deep moral beliefs by either oneself or other trusted individuals. As defined by Litz et al. (19), these potentially morally injurious experiences may involve perpetuating, failing to prevent, bearing witness to, or learning about actions that transgress deeply held moral beliefs. The concept of MI has gained traction in HCWs and PSP in recent years, but it continues to remain ill-defined in these populations (21, 22). Although empirical studies in the healthcare context remain limited, it is probable that existing relationships between MI and increased susceptibility to various mental health outcomes, including the emergence of posttraumatic stress disorder (PTSD), major depressive disorder, and increased suicidality among military (23–25) and public safety personnel (26), will extend to members of the healthcare professions.

Although the concepts of MD and MI share similarities, there remains ambiguity around their definitions (15, 27, 28). One approach proposed by Litz and Kerig (29) is to conceptualize MD and MI as existing on a continuum, with MD appearing on the less severe end of this spectrum. However, others have suggested that MD and MI result from different types of insults, with MD resulting from the long-term accumulation of damage from organizational oppression, while MI results from the immediate harm resulting from a single substantial act going against individual beliefs (30). Given the limitations of these definitions, it has been suggested to integrate and synthesize the concepts of MD and MI as one, to better explore moral stressors holistically (28). Accordingly, we have taken a similar approach to explore these concepts conjointly in the context of COVID-19. The goal of this review was not to delineate the conceptual similarities and differences between MD and MI, but rather to gain a better appreciation of the moral stressors faced by HCWs and PSPs during the pandemic, particularly given their potential relationship to negative mental health outcomes Indeed, HCWs working during the pandemic have reported symptoms of depression, anxiety, insomnia, and distress, up to 50.4, 44.6, 34.9, and 71.5%, respectively, in a sample of 1,257 HCWs, with frontline workers involved directly in the care of COVID-19 patients having a significantly higher risk of all symptoms (31). PSP have also reported anxiety and depressive symptoms during the pandemic, with those exposed to the virus reporting significantly higher alcohol use severity compared to their non-exposed counterparts (13).

Although the majority of interest is focused on shared circumstances that may give rise to MD/MI across the globe, unique circumstances across countries and continents during the COVID-19 pandemic exert the potential to expose HCWs and PSP to unique PMIDEs across different geographic regions. Together, these unique circumstances may include a country’s population density and healthcare capacity, along with cultural and social factors (e.g., civil society and trust in the healthcare system) that influence pandemic response (32, 33). In addition, HCWs and PSP may be differentially accustomed to the resource shortages experienced during the pandemic (34).

On balance, the extant literature suggests that HCWs and PSP appear susceptible to the development of MD and MI related to their pandemic service. Despite this knowledge, it is unclear to date which unique and shared PMIDEs across geographies may contribute to the development of MD and MI in the COVID-19 context. Accordingly, the purpose of this scoping review was to better identify and describe the existing literature examining COVID-19 related PMIDEs in HCWs and PSP on a global scale, with particular focus on shared and non-shared PMIDEs.

## Materials and Methods

Given the emerging and complex nature of MD and MI in HCWs and PSP during COVID-19, a scoping review approach was chosen to explore the nuanced and heterogeneous literature in this field (35).

This scoping review followed the five-step approach outlined by Arksey and O’Malley (36) and further built upon by Levac et al. (37). The five steps include: identifying the research question, identifying relevant studies, selecting studies, charting the data, and collating, summarizing, and reporting the results. The present study complies with the preferred reporting items for systematic reviews and meta-analyses extension for scoping reviews (PRISMA-ScR) checklist (38) (Supplementary Table 1).

### Identification of the Research Question

To gain a better understanding of the global context of MD and MI in HCWs and PSP during COVID-19, the research question was: ‘What are the shared and unique circumstances of HCWs and PSP during COVID-19 across the globe that are potentially associated with MD and MI?’

### Identification of Relevant Studies

The search terms were established through discussion with experienced researchers and clinicians within the field (MCM, KR), followed by the iterative drafting of the search strategy with an experienced librarian (LB) (Supplementary Data 1). A total of six databases (Medline, Embase, Global Health, CINAHL, Web of Science, and PsychInfo) were searched from January 1, 2020 to May 21, 2021 for articles that focused on HCWs (1) and select PSP (i.e., paramedics, firefighters, police officers, correctional officers, and emergency dispatchers), the COVID-19 context, and MD or MI. Handsearching of relevant studies and the references of included review articles were also performed.

### Selection of Studies

The eligibility criteria included: published articles that focused on HCWs (including but not limited to nurses, physicians, respiratory therapists, occupational therapists, physical therapists, physician assistants, psychologists, social workers, and supporting healthcare staff) and select PSP (paramedics, firefighters, police officers, correctional officers, and emergency dispatchers) during the COVID-19 pandemic. Articles were included if they provided an in-depth focus on circumstances that may lead to MD or MI. As MD and MI are not universal terms used across the world, articles that did not mention MD or MI were also included if they: (a) provided events that may be considered a PMIDE; (b) reported outcome(s) within the scope of MD or MI; and (c) provided probable or direct connections between the PMIDE (a) and outcome(s) (b). The specific criteria were informed by literature on MD and MI and are listed below:

(a) PMIDEs included events where HCWs or PSP performed, witnessed, or were placed in situations that forced them to engage in acts violating deeply held personal or professional moral beliefs or expectations (19, 27); experienced moral uncertainty due to constraints within or outside of their control (internal or external) (15); or experienced organizational betrayal (39, 40).
(b) Emotional, psychological, behavioral, social, spiritual/existential, or functional outcomes, including, but not limited to, moral emotions such as shame, guilt, and anger, betrayal, anhedonia, inward hostility, social alienation, loss of trust in self or others (20, 39), and mental health diagnoses such as PTSD or major depressive disorder (23, 25).
(c) Connections between PMIDEs and associated outcomes were determined by the reviewers using both the phrasing of the articles’ findings as well as statistical analyses connecting the PMIDE and outcome, if applicable.

Quantitative, qualitative, and mixed-method primary studies available in English were included, in addition to editorials and commentaries that provided primary data or explicit personal narratives. Review articles were appraised to identify additional relevant articles. Although the inclusion of articles written in any language would have been ideal, given resource limitations, and the complex nature of conceptualizing MD and MI, which may not be sufficiently understood using language translation software, a decision was made to focus on articles available in English. Given the global scope of the review, there were no limits placed on the geographical location of the study. Exclusion criteria included gray literature as well as published articles that were primarily focused on ethical analyses, policy recommendations, settings outside of healthcare and public safety, or which lacked an in-depth focus on MD or MI. Two reviewers (YX and JL) independently screened the full text of all articles against the eligibility criteria. Any conflicts in inclusion were resolved by a third reviewer (KR).

**FIGURE 1:**
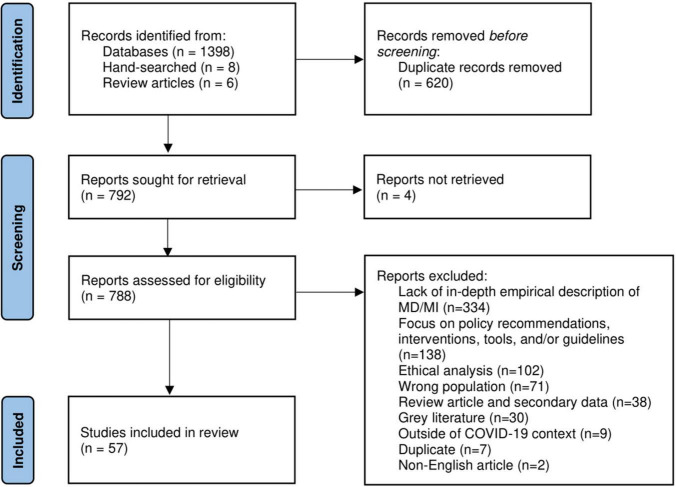
PRISMA flow diagram.

A total of 1,412 articles were identified from seven databases, handsearching, and references of review articles, of which 620 duplicates were removed (Figure 1). The full text of 792 articles was screened for eligibility, and 57 were included in this review. The full text of four articles was unable to be retrieved and was not screened for eligibility.

### Charting the Data

A data extraction form was jointly created by the research team and piloted by two reviewers (YX and JL). Charted data items included basic study characteristics (study type, country of origin, setting, study period, concept of interest, population of interest, aims of the study, and outcome measures), and the following MD- and MI-related data as defined above: (i) description of PMIDE(s) or the circumstances contributing to the PMIDE(s), (ii) outcomes within the scope of MD and MI, and (iii) direct connections between (i) and (ii). Determining the type of data that constituted the description of the PMIDE(s) (i) required a degree of interpretation by the reviewers given the complex nature of the concept of morals. Nonetheless, inclusion was guided by the definition of a PMIDE as described above. Factors, practices, and interventions described to protect against MD and MI were initially included in the data extraction form, but following further discussion, these items were excluded as many articles that reported this data failed to meet the study’s eligibility criteria.

### Collating, Summarizing, and Reporting the Results

Following Levac et al. (37), the following steps were taken at this stage: analyzing the data, reporting the results, and applying meaning to the results. To identify the key characteristics related to our concept of interest, basic qualitative coding was performed on the extracted data as suggested by Peters et al. (35). These data were duplicated onto a separate Excel spreadsheet and the three data items related to MD and MI, (i) to (iii), were coded by one reviewer (YX) into three distinct sets of codes using the conventional qualitative content analysis approach (41, 42). Within each set of codes, themes were formed by first grouping common codes, followed by subsequent grouping by meaning. A descriptive numerical summary of included articles was also conducted with respect to all study characteristics. To better understand the universality of the identified PMIDE themes, a geographical summary of the articles included in each theme, specifically focused on the geography of the studied population, was conducted. Geographic regions outlined by the World Health Organization (WHO) were used to define geography (43). Meaning derived from identified categories and their geographical distribution was iteratively generated and discussed amongst the team, with any discrepancies resolved through consensus.

## Results

**FIGURE 2:**
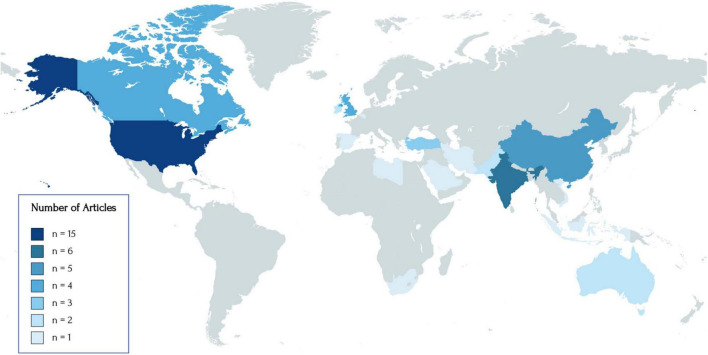
World map of the geographical distribution of included studies. The following geographies were represented: United States (*n* = 15), India (*n* = 6), China (*n* = 5), Canada (*n* = 4), United Kingdom (*n* = 4), Turkey (*n* = 3), Australia (*n* = 2), Ireland (*n* = 2), Lebanon (*n* = 2), Pakistan (*n* = 2), Belgium (*n* = 1), Indonesia (*n* = 1), Iran (*n* = 1), Libya (*n* = 1), Palestine (*n* = 1), Saudi Arabia (*n* = 1), South Africa (*n* = 1), Spain (*n* = 1), and Vietnam (*n* = 1). Two studies conducted with global participants and one study conducted on North American participants were not included in the following figure.

Of the 57 included articles, the majority were conducted on populations in the United States (*n* = 15), followed by India (*n* = 6), China (*n* = 5), United Kingdom (*n* = 4), Canada (*n* = 4), Turkey (*n* = 3), Australia (*n* = 2), Lebanon (*n* = 2), Pakistan (*n* = 2), Ireland (*n* = 2), and one study from Belgium, Indonesia, Iran, Libya, Palestine, Saudi Arabia, South Africa, Spain, and Vietnam. Two studies recruited participants globally (44, 45) and another study was focused on the North American context (46). The geographical distribution of included studies is illustrated in Figure 2. The majority of articles were published in 2020 (*n* = 31) and recruited participants or collected data in April (*n* = 15), May (*n* = 17), June (*n* = 18), and/or July (*n* = 10) of that year. About 35.1% of articles used surveys only (*n* = 20), 36.8% used interviews only (*n* = 21), 3.51% used focus groups only (*n* = 2), 1.75% used interviews and focus groups (*n* = 1), 3.51% used both surveys and interviews/focus groups (*n* = 2), and the remaining articles provided data from the authors’ personal experiences or from their discussions with peers (*n* = 11). All articles using surveys had a cross-sectional design. Five articles using surveys included questions with free-text responses. Demographic information for all included studies is presented in Table 1.

**TABLE 1:** Study characteristics of included articles.

Author	Region	N	Population(s) (n)	Setting	Research design	Recruitment/Data collection period	Study purpose
Ayyala et al. (46)	North America	251	Physicians (n = 251)	Pediatric radiology (hospital and remote)	Quantitative (cross-sectional, survey)	April–May 2020	Explore the sources of stress and anxiety for faculty in pediatric radiology during the early stage of COVID-19
Banerjee et al. (63)	India	172	Physicians (n = 172)	COVID-19-designated hospital	Qualitative (interview)	April–August 2020	Explore the adversities of HCWs and construct a conceptual framework of their psychological resilience.
Bayrak et al. (55)	Turkey	618	Nurses (n = 618)	Health institution	Quantitative (cross-sectional, survey)	May 2020	Explore the relationship between anxiety levels and the anger expression styles of nurses during the COVID-19 pandemic.
Billings et al. (91)	United Kingdom	28	Mental health professionals (n = 28)	NR	Qualitative (interview)	June 8 - July 23, 2020	Explore the experiences, views, and needs of United Kingdom mental health professionals during the COVID-19 pandemic.
Brophy et al. (47)	Canada	10	Nurses (n = 5) Personal support workers (n = 2) Administrative staff (n = 2) Cleaner (n = 1)	Long-term care home/Hospital	Qualitative (interview)	April–May 2020	Explore how HCWs are navigating the compromised healthcare system in Ontario while facing the increased risk and pressures of COVID-19.
Creese et al. (56)	Ireland	48	Physicians (n = 48)	Hospital	Qualitative (Interview)	June–July 2020	Explore the perceptions of doctors of their own mental and physical well-being during the first wave of COVID-19.
Ditwiler et al. (48)	United States	10	Physical therapists (n = 10)	NR	Qualitative (interview)	23 June–17 July 2020	Explore the experiences of physical therapists on the professional and ethical issues encountered during COVID-19.
Fawaz and Itani (74)	Lebanon	18	Nurses (n = 18)	Ground zero hospital	Qualitative (interview)	January 2021	Explore the psychological experience of Lebanese frontline nurses serving at ground zero hospitals.
Ffrench-O’Carroll et al. (60)	Ireland	408	Nurses (n = 273) Physicians (n = 71) Allied health professionals (n = 35) General support staff (n = 16) Managerial/administrative/ IT staff (n = 7)	Intensive care unit (adult and pediatrics)	Correspondence (quantitative (cross-sectional, survey))	7 May–5 June 2020	Explore the extent of psychological distress on staff working in pediatric and adult ICUs during COVID-19.
Gaucher et al. (49)	Canada	187	Physicians (n = 187)	Emergency department (general and pediatrics)	Quantitative/qualitative (cross-sectional, survey)	29 June–29 July 2020	Explore the experiences, concerns, and perspectives during the first wave of the pandemic.
Gunawan et al. (66)	Indonesia	17	Nurses (n = 17)	Hospital	Qualitative (interview)	March–June 2020	Explore the lived experiences of nurses combatting COVID-19.
Jia et al. (70)	China	18	Nurses (n = 18)	COVID-19-designated unit	Qualitative (interview)	February–March 2020	Examine ethical challenges encountered by nurses and their coping styles to ethical conflicts and dilemmas.
Kaelen et al. (58)	Belgium	44	Nurse-aid (n = 17) Nurse (n = 10) Occupational therapist or physical therapist (n = 9) Support staff (n = 8)	Nursing home	Qualitative (focus groups)	15 June–3 July 2020	Examine the psychosocial and mental health needs of nursing home residents during of the first wave of COVID-19 and how nursing home staff perceived their preparedness to address those needs.
Kanaris (90)	United Kingdom	NA	Healthcare workers (NA)	Intensive care unit	Commentary	NA	NR
Maraqa et al. (96)	Palestine	357 (quantitative), 15 (qualitative)	Quantitative: nurses (n = 161), physicians (n = 156), others (n = 40); Qualitative: physicians (n = 7), nurses (n = 6), lab technician (n = 1), radiology technician (n = 1)	Hospitals and public health centres	Mixed methods (cross-sectional, survey; interviews)	Quantitative: 2*^nd^* month of COVID-19 outbreak in Palestine Qualitative: 3*^rd^* month of COVID-19 outbreak in Palestine	Explore healthcare workers’ willingness to work and the associated factors, in addition to the ethical dilemmas during COVID-19.
Al Muharraq (77)	Saudi Arabia	215	Nurses (n = 215)	Hospital	Quantitative (survey, cross-sectional)	Aug-20	Explore the psychological impact of COVID-19 and coping strategies in frontline nurses working in Jazan, Saudi Arabia.
O’Neal et al. (44)	Global (primarily United States, Kenya, Canada)	839	Physicians (n = 540) Nurses (n = 111) Mental healthcare provider (n = 52) Physician assistant (n = 11) Paramedic or EMT (n = 10) Laboratory technician (n = 3) Respiratory therapists (n = 2) Others (n = 49)	Various settings^a^	Quantitative (cross-sectional, survey)	19 May–30 June 2020	Explore the scope and specifics of moral distress and HCWs perception of risk during COVID-19 and generate discussion around ethical resource allocation.
Patterson et al. (54)	United States	34	Medical family therapists and trainees (NR) Physicians and residents (NR)	Family medicine clinic	Qualitative (cross-sectional, survey)	May–June 2020	Explore moral distress in clinicians working in a family medicine setting.
Rao et al. (51)	United States	50	Physicians (n = 22) Nurses (n = 21) Respiratory therapists (n = 2) Paramedics (n = 4) Emergency medical technician (n = 1) Physician assistant (n = 1)	Safety net hospital	Qualitative (interview)	22 April–8 July 2020	Examine factors driving distress and motivation in interdisciplinary clinicians caring for patients with COVID-19.
Şahin and Kulakaç (59)	Turkey	356	Nurses (n = 210) Physicians (n = 51) Emergency medical technicians/ Anaesthesia technician/Cleaning personnel (NR) Others (n = 27)	Hospital	Quantitative (cross-sectional, survey)	10-15 May 2020	Explore anxiety levels of healthcare workers during COVID-19 and associated factors.
Silverman et al. (83)	United States	31	Nurses (n = 31)	Academic Medical Centres (Acute Care)	Qualitative (focus groups/interviews)	April–June 2020	Explore the causes of moral distress in nurses caring for COVID-19 patients and identify strategies to cope with threatened moral integrity.
Sukhera et al. (84)	Canada	22	Resident physician (n = 17) Faculty member (n = 5)	Hospital	Qualitative (interview)	April–June 2020	Explore how residents perceive moral distress in relation to structural stigma during COVID-19.
Tate (87)	United States	NA	Physician (n = 1)	Pediatric palliative care	Commentary	NA	NR
Wanigasooriya et al. (61)	United Kingdom	2638	Nurses (n = 775) Physicians (n = 460) Others*^b^* (n = 1403)	Hospital (acute general and mental health)	Quantitative (cross-section, survey)	5 June–31 July 2020	Evaluate rates of clinically significant symptoms of anxiety, depressive and post-traumatic stress disorder and associated exposures and characteristics in HCWs following the first COVID-19 peak.
Whitehead et al. (88)	United States	19	Nurse managers (n = 19)	Healthcare organizations	Qualitative (interview)	NR	Examine the moral distress experience of nurse managers.
Wiener et al. (11)	United States	207	Nurses (NR) Physicians (NR) Child life specialists (NR) Social workers (NR) Chaplains (NR) Psychologists (NR)	Pediatric palliative care	Quantitative/qualitative (cross-section, survey)	1 May–26 June 2020	Explore the impact of COVID-19 on end-of-life care and the approach taken by providers toward bereavement care in pediatric palliative care.
Yıldırım et al. (62)	Turkey	17	Nurse (n = 17)	Hospital (COVID-19 unit)	Qualitative (interview)	27 May–25 August 2020	Explore the experiences of nurses working during the onset of the COVID-19 outbreak in Turkey.
Wang et al. (99)	China	3006	Physicians (n = 2423) Nurses (n = 583)	Hospital	Quantitative (cross-sectional, survey)	27 March–26 April 2020	Examine prevalence and correlates of moral injury in physicians and nurses in during COVID-19.
Ananda-Rajah et al. (69)	Australia	569	Physicians (n = 331) Nurses (n = 188) Allied health professionals (n = 25) Paramedics (n = 13) Administrative staff (n = 7) Midwives (n = 5)	NR	Qualitative (open-letter with one free-text response question)	August–October 2020	Explore the working condition and issues faced by HCWs during COVID-19.
Benzel (52)	United States	NR	Healthcare workers (NR)	Hospital	Commentary	NA	NR
Butler et al. (85)	United States	61	Physicians (n = 50) Nurses (n = 10)	Academic institutions, private institution, other	Qualitative (interview)	April–May 2020	Describe the perspectives and experiences of clinicians involved in the institutional planning for resource limitation or patient care during COVID-19.
Cai et al. (71)	China	534	Nurse (n = 248) Physician (n = 233) Medical technician (n = 48) Hospital staff (n = 5)	Hospital	Quantitative (cross-sectional, survey)	January–March 2020	Explore the psychological impact and coping strategies of frontline healthcare staff in the Hunan province.
Cheng and Li Ping Wah-Pun Sin (89)	United Kingdom	NA	Physician (n = 2)	Palliative care	Commentary	NA	NR
Cheriyan and Kumar (94)	India	286	Medical residents (n = 286)	Urology	Editorial (quantitative (cross-sectional, survey)	21 June–11 July 2020	Explore the impact of COVID-19 on the training and academics, clinical work, and personal life of urology residents.
Collado-Boira et al. (57)	Spain	62	Medical students (n = 33) Nursing students (n = 29)	Hospital	Qualitative (Interview)	March–April 2020	Explore the perceptions of nursing and medical students during COVID-19.
Dewar et al. (86)	Canada	165	Physicians (n = 165)	Hospital	Quantitative/Qualitative (cross-sectional, survey)	3–13 April 2020	Explore the preparedness and attitudes of physicians on resource allocation decisions.
Dhar and Wani (67)	India	NA	Surgeons (NR)	NR	Commentary	NA	NR
Do Duy et al. (82)	Vietnam	61	Nurses (45) Clinicians (7) Others (9)	Hospital	Editorial (quantitative, cross-sectional, survey)	26–29 April 2020	Measure the stigma experienced by HCWs after 3 weeks of quarantine and its association with mental health problems.
Elhadi et al. (98)	Libya	745	Physicians (NR) Nurses (NR)	Hospital	Quantitative (cross-sectional, survey)	18–28 April 2020	Measure the prevalence of anxiety and depression in HCWs during COVID-19 and the civil war in Libya.
Evans et al. (92)	Australia	NA	Healthcare workers (NA)	Palliative care	Ethics Rounds	NA	NR
Fawaz and Samaha (97)	Lebanon	13	Nurses (n = 9) Physicians (n = 4)	Hospital	Qualitative (interview)	NR	Explore psychosocial effects of being quarantined following COVID-19 exposure in HCWs.
George et al. (65)	India	64	Physicians (n = 20) Nurses (n = 14) Field staff (n = 14) Allied health professionals (n = 10) Others (n = 6)	Urban slum	Mixed methods (cross-section survey, interview and focus group)	First 40 days of pandemic	Explore dilemmas, mental stress, adaptive measures, and coping strategies in healthcare teams providing healthcare services in urban slums.
Iheduru-Anderson (50)	United States	28	Nurses (n = 28)	Hospital (acute care)	Qualitative (interview)	15 May–20 June 2020	Explore experiences of nurses working with limited PPE during COVID-19.
Kling (81)	South Africa	NA	Physician (n = 1)	Hospital	Commentary	NA	Explore the duty of care and side-line guilt during COVID-19.
Koven (80)	United States	1	Physician (n = 1)	Hospital	Commentary	NA	NA
Li et al. (73)	China	150	Nurse (n = 107) Physician (n = 43)	Hospital	Quantitative (cross-sectional, survey)	1-20 February 2020	Examine the relationships between sociodemographic characteristics and anxiety and depression in frontline medical workers.
Liu et al. (72)	China	13	Nurses (n = 9) Physicians (n = 4)	COVID-19-designated Hospital	Qualitative (interview)	10-15 Feb 2020	Explore experience of HCWs during early stages of outbreak
Mehra et al. (64)	India	88	Medical residents (n = 23) Paramedical staff (n = 23) Faculty members (n = 17) Medical officers (n = 3) Security staff (n = 3) Nursing staff (n = 1) Others (n = 18)	Tertiary care center	Quantitative (cross-sectional, survey)	April–May 2020	Evaluate the prevalence of psychological issues in HCWs working in a tertiary care center.
Mohindra et al. (68)	India	NR	Frontline healthcare providers (NR)	Tertiary hospital	Qualitative (interview)	NR	NR
Noreen et al. (76)	Pakistan	250	Consultant (n = 40) Medical officers (n = 70) Faculty (n = 53) Resident (n = 47) House officer (n = 40)	NR	Quantitative (cross-sectional survey)	NR	Explore the factors impacting psychological health and coping strategies of healthcare professionals during COVID-19.
Raza et al. (75)	Pakistan	12	Physicians (n = 7) Nurses (n = 5)	COVID-19-designated hospital	Qualitative (interview)	6-14 April 2020	Explore factors that impede healthcare providers to effectively treat COVID-19 patients.
Reuben (78)	United States	NA	Physician (n = 1)	NR	Perspective	NA	NR
Rezaee et al. (93)	Iran	24	Nurses (n = 24)	Educational and medical centers	Qualitative (interview)	September–October 2020	Explore perceived ethical challenges of nurses caring for patients with COVID-19.
Shanafelt et al. (7)	United States	69	Physicians (NR) Nurses (NR) Advanced practice clinicians (NR) Residents (NR) Fellows (NR)	NR	Perspective (focus groups)	First week of the COVID-19	Explore the concerns of healthcare professionals, the messaging and behaviours they need from their leaders, and the sources of support they believe would be most useful for them.
Tsai (79)	United States	1	Physician (n = 1)	Hospital	Commentary	NA	NA
Viswanathan et al. (53)	United States	130/57*^c^*	Attending physicians (n = 40) Residents (n = 40) Nurses (n = 50) Unknown (n = 57)	Hospital	Perspective	Beginning March 28 2020	Describe experience of providing peer support groups and individual counseling to HCWs that focus on issues and emotions related to their work during COVID.
Zolnikov & Furio (45)	Global (primarily United States)	31	Nurses (n = 14) Physician (n = 3) Police offers (n = 3) Firefighters and paramedics (n = 3) Firefighter and emergency medical technician (n = 1) Paramedics (n = 1) Others*^d^* (n = 6)	NR	Qualitative (interview)	NR	Explore the stigma toward first responders during COVID-19 and its associated consequences on mental health.

*^a^Academic medical center (n = 270), community private health system (n = 234), government health system (n = 113), long-term care or assisted living (n = 7), out-of-hospital/ambulance (n = 9), prison or other detention health system (n = 4), veterans health administration system (n = 16), other (n = 114).*

*^b^Non-clinical staff, allied health professions, and other.*

*^c^130 individuals participated in the support group sessions, 57 individuals participated in the individual mental health care sessions.*

*^d^Behavioral therapist (n = 1), orthodontist (n = 1), dialysis technician (n = 1), medical surgery technician (n = 1), data specialist (n = 1), tech (n = 1). NR, not reported.*

A total estimated sample size of 12,488 participants is represented by all articles that provided a sample size (*n* = 46). The sample size of these articles ranged from 10 to 3,006 participants (median of 63 participants). All articles primarily focused on HCWs, and in articles specifying the occupation of HCWs, the most common populations studied were physicians (37 of 57 studies) and nurses (34 of 57 studies). Data on the PSP population were extremely limited, including very small samples of paramedics, emergency medical technicians, police officers, and/or firefighters, aggregated with the data of HCWs within six studies. Given the lack of literature on PSP, the findings presented here are based almost exclusively on the experience of HCWs.

The included articles focused on exploring the experiences, challenges, and stressors faced by HCWs, their coping strategies, and psychological outcomes. Two studies used a validated measure of MI and MD respectively (Supplementary Data 2).

Six overarching themes describing the circumstances associated with PMIDEs were identified. The themes were: (1) Risk of contracting or transmitting COVID-19; (2) Inability to work on the frontlines; (3) Provision of suboptimal care; (4) Care prioritization and resource allocation; (5) Perceived lack of support and unfair treatment by their organization; and (6) Stigma, discrimination, and abuse. The studies included under each PMIDE category as well as their geographic distribution are provided in Supplementary Table 2.

### Risk of Contracting or Transmitting COVID-19

Fear of contracting COVID-19 and infecting family, colleagues, and/or patients, was described by HCWs in 34 articles (59.6%). These studies collectively had the greatest global spread of all PMIDE categories, representing data from participants in the Americas (*n* = 10) (7, 11, 47–54), Europe (*n* = 8) (55–62), South-east Asia (*n* = 6) (63–68), Western Pacific (*n* = 5) (69–73), Eastern Mediterranean (*n* = 4) (74–77), and globally (*n* = 1) (44). Feelings of fear (58, 68, 72, 73, 75–77), anxiety (7, 44, 47, 52, 73), guilt (50, 54, 56, 63, 65), and depression (73) were commonly described throughout the included studies. The following sections highlight the strenuous moral decisions HCWs often faced in balancing professional duties with personal and familial safety.

The fear associated with contracting and transmitting COVID-19 was especially prominent among HCWs who had additional vulnerabilities to infection due to an underlying health condition (44, 54, 55, 59), or who lived with children, older adults, or individuals with other vulnerabilities (44, 50, 55, 65, 72) (Turkey, India, Global, China, and United States). Feelings of guilt were commonly reported due to feeling as though one was putting one’s family and colleagues at risk of infection or in relation to not wanting to be on the frontlines because of an underlying health condition (50, 54, 56, 63, 65) (United States, Ireland, and India). Concerns around infection sometimes led to taking time off from work (47) (Canada).

Exacerbating these concerns were shortages in PPE and the uncertainty around what constituted sufficient PPE (48, 54, 74) (United States and Lebanon). In a global cross-sectional study (44), 62.9% of participants reported a PPE shortage, which was present in nearly all settings and was reported similarly between physicians and other HCWs. Regional studies replicated these concerns around PPE supply. About 49% of physicians working in the emergency department reported concerns around lack of PPE, with 31% occasionally providing care without appropriate PPE (49) (Canada). Similarly, nursing home staff have reported insufficient or a complete lack of appropriate PPE for weeks (58) (Belgium). The lack of adequate PPE sometimes forced HCWs to clean and reuse their equipment (66) (Indonesia). In relation to mental health outcomes, HCWs who lacked access to adequate PPE experienced significantly greater symptoms of generalized anxiety disorder, major depressive disorder, and PTSD (61) (United Kingdom). Additionally, workers in the adult intensive care unit (ICU) reported significantly higher levels of stress with respect to equipment and staff shortages than did workers in the pediatric ICU (60) (Ireland).

### Inability to Work on the Frontlines

Thirteen articles (22.8%) described situations where some HCWs were unable to work to the same extent as their colleagues on the frontlines, representing samples from the Americas (*n* = 8) (46, 50, 52–54, 78–80), South-east Asia (n = 2) (67, 68), and one article each from Africa (81), Europe (56), and Western Pacific (82). These situations were described by some HCWs to be associated with feelings of guilt and shame (50, 52, 53, 56, 78, 81, 82).

Feelings of guilt were reported by HCWs who felt that they did not have the same level of exposure or risk as their colleagues, including surgeons (52, 67) (United States, India), radiologists (46) (North America), students (54) (United States), and other HCWs not assigned to work in-person, or who chose to switch to telemedicine due to underlying comorbidities (50, 54, 79, 80) (United States). Similar feelings were reported by HCWs who were required to isolate and quarantine following exposure or infection by COVID-19 (53, 56, 82) (Ireland, Vietnam, and United States). These feelings have been termed “sideline guilt” (78, 81) (United States, South Africa). In addition, consistent with the concept of survivor guilt, some HCWs have reported feelings of guilt and sorrow in relation to recovering from illness or not becoming ill while some of their colleagues died (50) (United States).

### Provision of Suboptimal Care

The uncertainty and ambiguity experienced by HCWs around patient care were also pervasive. Given an early lack of clinical guidelines and evidence surrounding COVID-19, HCWs described difficulty deciding on and communicating an appropriate therapeutic course of action and a realistic prognosis in many cases (48, 51, 53, 81, 83) (United States, South Africa). Thirty-three articles (57.9%) discussed the idea of providing suboptimal care, representing diverse samples across the globe, including the Americas (*n* = 15) (7, 11, 48–54, 83–88), Europe (*n* = 9) (56–58, 60–62, 89–91), Western Pacific (*n* = 3) (70, 72, 92), Eastern Mediterranean (*n* = 2) (74, 93), South-east Asia (*n* = 2) (67, 94), and one from Africa (81) and another with a global scope (44). From these articles, three subcategories were formed: “PPE negatively impacting care”; “Inability to provide a good death”; and “Unprepared for novel tasks.” HCWs drew connections between providing suboptimal care or seeing patients dying alone and feelings of guilt (50, 54, 74), sorrow (11, 51), worry (70, 88), and powerlessness (72, 84).

Concerns about the quality of care and support provided to patients were commonly reported (48, 49, 54, 83, 88, 93) (United States, Canada, Iran). HCWs often reported feeling responsible for the patients’ outcomes and experiencing intrusive thoughts and feelings of guilt, despite following institutional recommendations (54, 74, 83, 85) (United States, Lebanon). Being unable to observe patients achieve the outcomes that they would typically achieve prior to the pandemic led to feelings of hopelessness and lack of control (48, 53, 72) (United States, China). These experiences were also reported in HCW managers who described their own MD when managing and supporting staff, in addition to the MD experienced by their team (88) (United States). Conflicts between colleagues in patient care plan decisions and witnessing inadequate provision of care were described as a source of MD (83) (United States). Witnessing the disproportionate harm to stigmatized groups impacted by restrictive services and policy decisions, including limited access to culturally and linguistically appropriate services and mental health and addiction care needs, also led to considerable MD (84) (Canada).

#### PPE Negatively Impacting Care

Eight studies across the Americas (*n* = 4) (49, 51, 53, 83), Europe (*n* = 2) (58, 89), South-east Asia (*n* = 1) (67), and Western Pacific (*n* = 1) (70), described how the use of PPE by HCWs acted as a significant physical and emotional barrier to patient care. The use of PPE and the need for distancing made it difficult to verbally communicate with patients (49, 58, 89) (United Kingdom, Canada, Belgium) and convey emotion, with some HCWs reporting that the use of PPE increased fear in patients (49, 51, 53, 70, 89) (Canada, United States, China, United Kingdom). These challenges depersonalized care and made it difficult for HCWs to develop trust and rapport with patients (51, 53, 86, 89) (United States, Canada, United Kingdom). Moreover, the additional time required to don and doff PPE sometimes led to treatment delays and failures (49, 70, 83) (Canada, China, United States).

#### Inability to Provide a Good Death

Ten articles described how HCWs were unable to provide patients with a “good death” given the circumstances of the pandemic. These articles represented samples from the Americas (*n* = 6) (11, 53, 83, 86–88), Europe (*n* = 2) (89, 90), Eastern Mediterranean (*n* = 1) (93), and Western Pacific (*n* = 1) (92).

Healthcare workers reported how it was overwhelming to see patients die alone without their loved ones (53, 83, 90, 93) (United States, United Kingdom, Iran), and how visitation policies added to the distress experienced by families (49) (Canada). Moreover, the delivery of bad news to families by telephone was described as limiting the humane aspect of being a care provider (89) (United Kingdom). HCWs described feeling responsible for allowing patients to die alone and experienced ruminating thoughts related to these experiences (90) (United Kingdom). These challenges were especially prominent in the end-of-life care of pediatric patients where visitation policies often allowed only a single parent to be with a child until he or she was reaching the end of life (11, 60) (Ireland, United States). HCWs enforcing these policies described a feeling of participating in something “evil” which went against their morals (60) (Ireland). In the United States, these providers further described morally distressing experiences where they felt constrained from doing what they believed to be ethically appropriate (e.g., being instructed to separate a dying infant from the mother), observed potentially traumatic events that they were unable to change (e.g., informal funerals in a family’s yard), and experienced ambivalence about the morals of their actions (e.g., making decisions around employee furloughs, salaries and hiring) (11). Witnessing or learning about colleagues providing inappropriate end-of-life care for patients and their families was also described as increasing MD in HCWs (83, 88) (United States).

#### Unprepared for Novel Tasks

Another factor that may have limited HCWs’ ability to provide optimal care was the lack of preparation for novel tasks beyond the scope of their role. Nineteen articles described the events of participants from the Americas (*n* = 6) (7, 48, 49, 51, 54, 83), Europe (*n* = 8) (57, 58, 60–62, 89–91) South-east Asia (*n* = 2) (67, 94), Western Pacific (*n* = 2) (70, 72), and globally (*n* = 1) (44). Many HCWs were redeployed during the pandemic to assignments where they took on unfamiliar roles—junior doctors were now regularly breaking bad news (89) (United Kingdom), allied health professionals assisted in kitchen and nursing tasks (58) (Belgium), and pediatric ICU providers cared for adult patients (90) (United Kingdom). Many HCWs felt unprepared to perform their tasks (49, 57, 70, 83, 94) (Canada, Spain, China, United States, India), which included the management of psychological issues experienced by patients (70) (China) and frontline staff (91) (United Kingdom), as well as palliative care provision (86) (Canada). Some HCWs were also working in intensive care wards and handling infectious diseases for the first time (72, 83) (China, United States). In a United Kingdom study, redeployment was found to be significantly associated with PTSD symptoms, but not with symptoms of generalized anxiety disorder or major depressive disorder (61). Moreover, certain populations were differentially impacted by redeployment. When compared to their adult ICU counterparts, pediatric ICU workers reported significantly more stress toward being redeployed and treating patients outside of their trained role (60) (Ireland). Nurses also experienced significantly more stress than doctors and other professionals with respect to being redeployed and caring for patients outside of their trained role (60) (Ireland).

### Care Prioritization and Resource Allocation

Given the influx of patients infected by COVID-19 as well as limited material and human resources, HCWs were often faced with decisions surrounding care prioritization and resource allocation. Twelve articles described these challenges, representing HCWs from the Americas (*n* = 6) (11, 48, 51, 83, 85, 86), Europe (*n* = 2) (89, 91), Western Pacific (*n* = 2) (72, 92), Eastern Mediterranean (*n* = 1) (74), and globally (*n* = 1) (44). These experiences were sometimes associated with feelings of fear (85), anxiety (86), sadness (86), or guilt (91).

Many articles described the very high levels of fear and worry that HCWs experienced in relation to rationing resources, particularly critical care resources, and delaying elective treatment (51, 85, 89) (United States, United Kingdom). A survey in Canada found that the most common feelings experienced by physicians when making ventilator allocation decisions were sadness (24%) and guilt (19%) (86). Many HCWs felt that they did not receive adequate training to make these decisions; 53.9% of HCWs in a global study reported that they “disagreed” or “strongly disagreed” with statements related to having received sufficient training to allocate limited resources (44). Moreover, experiencing a higher exposure to moral dilemmas, which may encompass difficult allocation decisions, was associated with significantly higher symptoms of generalized anxiety disorder, major depressive disorder, and PTSD (61) (United Kingdom). Mental health professionals struggled with the need to prioritize psychological support as they were sometimes required to prioritize support of staff over support of vulnerable patients (91) (United Kingdom).

### Perceived Lack of Support and Unfair Treatment by Their Organization

A highly pervasive theme among HCWs was the lack of support or unfair treatment experienced by their institutions during the pandemic. A total of 16 articles discussed these experiences, which accounted for a global spread of countries across the Americas (*n* = 8) (7, 47, 48, 51–53, 88, 95), South-east Asia (*n* = 3) (63, 64, 66), Europe (*n* = 2) (58, 62), Eastern Mediterranean (*n* = 2) (75, 96), and Western Pacific (*n* = 1) (69). These experiences were often associated with feelings of anger (47, 50, 53, 58, 64, 69, 74) and betrayal (47, 50, 66, 69). Within this PMIDE category, a variety of subcategories were present: “Lack of adequate benefits”; “Decisions placing HCWs’ health at risk”; and “Lack of communication and transparency.”

#### Lack of Adequate Benefits

Many articles spoke to the inadequate support and benefits that some HCWs experienced during their work on the frontlines. In a study on Indian HCWs, the three highest unmet needs reported by participants were flexible work policies (88%), medical/insurance benefits (70%), and administrative understanding (60%) (63). HCWs were also concerned about the support that would be provided by their organization for their personal and familial needs, including the support that would be provided if they contracted COVID-19 (7) (United States). A study in the United States reported that many nurses lacked paid leave during their mandatory 2- to 3-week quarantines, which fostered a sense of betrayal toward their organization (50).

#### Decisions Placing HCWs’ Health at Risk

Healthcare workers commonly reported experiences of their institution placing their lives at risk and expressed concerns about workplace health and safety measures (63, 64, 69, 75) (India, Australia, Pakistan). In a study on Australian HCWs, PPE safety guidelines were inadequate and dictated by the resources available, rather than the safety of the staff (69). Other HCWs felt that financial resources and staff decisions were not allocated according to true need but were instead gauged using poor metrics that failed to account for on-the-ground realities (88) (United States). Some HCWs reported that their organization only improved the quality of PPE after providers were infected and became critically ill (69). Despite the inadequate PPE provided by their organization, many HCWs reported being shamed or facing repercussions such as work suspension for requesting or using personal supplies of PPE that was of better quality than that provided by their workplace (50, 69) (United States, Australia). These experiences were associated with a loss of trust in leadership, feelings of anger, frustration, and betrayal toward their organization for not protecting them, and thoughts of leaving the profession (50, 51, 58, 64, 69) (United States, Belgium, India, Australia). Similar concerns were found regarding the condition of COVID-19 wards, which sometimes lacked the necessary measures and facilities to ensure patient and staff safety (75) (Pakistan). In addition, staffing shortages were common among some HCWs and were described as a source of MD that overburdened staff and placed patients’ health at risk (88) (United States). HCWs also described feeling as though their institution did not care about their lives and they felt like a “pawn in a chess game” or a “machine” forced to face the situation regardless of the protection available (50, 53, 58, 62) (United States, Belgium, Turkey). Feeling vulnerable, dispensable, and abandoned were also described by some HCWs (51, 62, 88) (United States, Turkey). These feelings were not directed exclusively toward their institution, but at times toward the public which did not adhere to regulations, toward the government perceived as making poor decisions, and toward infectious disease experts who were not responsive to the concerns on the frontlines (47, 58, 69) (Canada, Belgium, Australia).

#### Lack of Communication and Transparency

Many issues were reported regarding the institutional transparency of decision-making processes and interactions with the public. HCWs reported a lack of consultation and inadequate communication with respect to the development of organizational policies (48, 58, 62, 69, 88) (United States, Belgium, Turkey, Australia). Some HCWs felt that their employers were not honest with the public regarding the situation at their institutions, communicating false scenarios of sufficient PPE and adequate employee safety (50) (United States). These issues appear to have been further exacerbated by reports of institutions silencing their workers’ concerns. Some HCWs were told to not express their concerns to the public, with some fearing or experiencing disciplinary action for speaking up (47, 50, 52, 66) (Canada, United States, Indonesia).

### Stigma, Discrimination, and Abuse

A total of 13 articles discussed the stigma that HCWs experienced during the COVID-19 pandemic. Unlike other categories, the geographic distribution of these articles was outside of the Americas, with the exception of one article (47) and included: South-east Asia (*n* = 4) (63, 65, 66, 68), Eastern Mediterranean (*n* = 3) (93, 97, 98), Western Pacific (*n* = 2) (82, 99), Europe (*n* = 2) (58, 62), and one global (45). These experiences were described by some HCWs alongside feelings of anger or frustration (98), betrayal (99), isolation (45, 63), depression (82, 97), and anxiety (82, 97). Many HCWs perceived that they were stigmatized by others (63, 68, 98) (India, Libya), especially workers on the COVID-19 unit (97) (Lebanon). This stigma was reported in 71% of Indian HCWs (63) and 31% of Libyan physicians (98) in two studies respectively. Other studies described experiences where family, friends, or colleagues rejected or treated HCWs as virus carriers (45, 62, 66, 82, 93, 97) (Global, Turkey, Indonesia, Vietnam, Iran, Lebanon). These events were associated with feelings of anger and pain in HCWs (62, 66, 97) (Turkey, Indonesia, Lebanon), as well as a reduction in spirits and motivation (58) (Belgium). The stigma faced by HCWs sometimes resulted in stigma toward their entire family (93) (Iran). In a study from Vietnam, 9.84% of HCWs felt blamed by their relatives or friends, and 39.34% reported that people were talking about them behind their backs (82). Another study found that stigma was significantly associated with both depressive and anxiety symptoms (98) (Libya).

Given the stigma experienced by frontline workers, some HCWs and PSP reported that they wished to hide their professional identity from others (45, 47, 65) (Global, Canada, India). These experiences were further amplified by negative portrayals of HCWs in the media (63) (India) and discriminatory actions against HCWs (63, 65, 82) (India, Vietnam). Two studies in India reported HCWs who were evicted from their apartments or asked to leave their job and residence by their communities (63, 65). Moreover, articles from Canada, Libya, and China (47, 98, 99) reported violence against HCWs by patients or patients’ family members. In a study of 745 Libyan HCWs, 52.3% reported experiencing abuse from patients or their relatives, with 45.8% facing three or more episodes of abuse, and 14.6% reporting physical abuse (98). In relation to anxiety and depressive symptoms, only verbal abuse was significantly associated with anxiety in these HCWs (98).

## Discussion

The present scoping review sought to provide a preliminary snapshot of PMIDEs encountered by HCWs and PSPs globally during the COVID-19 pandemic. A total of 57 articles were included, which focused almost exclusively on HCWs. Given the dearth of literature surrounding PSP, PMIDEs specific to this population were not identified, and further research is strongly needed to better understand the PMIDEs this population may be encountering during the pandemic. Overall, the identified PMIDE categories in HCWs cover a broad spectrum of moral dilemmas (Supplementary Table 3), including the need to manage competing responsibilities to patients, colleagues, oneself, or loved ones, uncontrollable factors limiting one’s ability to fulfill their duty to patients or colleagues, and risking one’s life for organizations or the public who treat them unfairly.

Although the empirical literature on MI in HCWs remains limited, insight can be drawn from the field of MD, which originated in the healthcare setting (14). Here, many of the major factors associated with MD described prior to the COVID-19 pandemic (14, 100, 101) largely parallel the PMIDE categories described in this review. For instance, the provision of care not in patients’ best interests (“Suboptimal care”), the emergence of organizational factors conflicting with patient care needs (“Care prioritization and resource allocation decisions”), and the presence of organizational hierarchies and a lack of administrative support (“Perceived lack of support and unfair treatment by their organization”). As described in numerous articles, the novel challenges of the COVID-19 pandemic, including human and resource supply limitations and the uncertainty around adequate infection control, triage, and treatment protocols for both frontline HCWs and leadership alike, likely exacerbated many of these factors. Finally, additional PMIDE categories specific to the COVID-19 context were identified in this review, including the “Risk of contracting or transmitting COVID-19,” “Inability to work on the frontlines,” and “Stigma, discrimination, and abuse.” Many of these categories are consistent with recent commentaries and editorials in the literature (102–109).

Overall, most categories of PMIDEs appear to be largely global in nature, encompassing diverse samples across the globe (Supplementary Table 2). However, given the uneven distribution of articles across geographies and the small sample sizes and number of articles available, further research is required to confirm these preliminary findings. In particular, the “Risk of contracting or transmitting COVID-19” encompassed the greatest distribution of articles globally, spanning over seven countries (Supplementary Table 2). The seemingly global nature of this PMIDE category may not be surprising as this phenomenon underlies the basic human need to survive and maintain the wellbeing of oneself, one’s family, and community members. Similar experiences have also been described by HCWs during past pandemics (110).

During the COVID-19 pandemic, the conflict that arose between HCWs’ duty to their patients and their duty to their families also emerged strongly as a source of moral suffering (103) and MI (109). One risk factor for MI included the loss of life of vulnerable individuals (111), elucidated further by the heightened feelings of fear, guilt, and anxiety reported by HCWs regarding the possibility of infecting children, older persons, and other vulnerable populations through their workplace. Additional factors impacting concerns around the infection in HCWs included their specific profession and amount of experience (61, 72), as well as reciprocal concerns from their family members (112). As described by Williamson et al. (111), insights gained earlier in military contexts may be helpful in remediating these mental health impacts of PMIDE exposure globally among HCWs.

Another PMIDE category that emerged as global in scale involved a “Perceived lack of support and unfair treatment of HCWs by their organization.” This category highlights the inadequate protection and support, as well as hostility, that some HCWs encountered from their organizations. Taken together, these findings may represent the institutional betrayal associated previously with MD (39) and MI (114), where institutional betrayal has been described as a violation of trust by an organization toward an individual who identifies with it (39, 115). Here, it is possible that COVID-19 has exacerbated and highlighted similar issues that existed pre-pandemic (116). As described in the articles included in this review, betrayal may take various forms in the healthcare setting, including inadequate workplace protections, disregard for HCW and patient needs, and gaslighting (39). These experiences have been linked to feelings of anger or frustration and thoughts of leaving the profession, and are thought to exacerbate the physical and psychological impacts of stressful events (39, 114, 117). For example, a recent study on betrayal-based MI during the COVID-19 pandemic indicated that HCWs commonly reported feeling abandoned and treated as dispensable by leadership, who were perceived as detached and dishonest at times (114). Interestingly, the study suggests that the behavior of the leadership team following acts of betrayal may be as impactful as the act itself. In the same study, HCWs reported that the lack of accountability, recognition, and apology, significantly influenced their experience of distress and burnout (114).

By contrast, the PMIDE category of ‘Stigma, discrimination, and abuse’ showed the least global spread among included articles. Despite North American articles accounting for the greatest proportion of studies included in this review (20 of 57 studies), 85% of the articles included in this category were outside North America. Although these findings may appear to highlight the uniqueness of this type of PMIDE in certain regions, it nonetheless remains possible that this issue is underreported or understudied in other regions across the globe. Here, a cross-sectional study evaluating the attitudes of non-HCWs in the United States and Canada found that over a quarter of respondents felt that severe restrictions should be placed on the freedom of HCWs, which included actions keeping them isolated from their communities and families (113). In one global study on COVID-19-related stigma, harassment, and bullying experienced by HCWs and non-HCWs, these experiences were reported to be the highest in Sub-Saharan Africa (14.0%), South Asia (10.7%), and North America (10.6%), with the latter two also containing the highest proportion of HCW respondents (118). Further research is urgently needed to better understand the context and severity of these experiences, which may or may not differ significantly across geographies. Potential factors that may impact these experiences regionally include the presence of a culture of blame within the society (119) and differences in how HCWs are portrayed by the media (120). When comparing different cultural responses to illness during the COVID-19 pandemic, one study found that individuals from China were more likely to behave aggressively toward doctors compared to individuals from the United States, who were more likely to direct their blame toward the health system (119). Finally, the media may play a role in perpetuating violence against HCWs, as noted by reports of misleading journalism in India (120).

The present scoping review provides insight into the PMIDEs reported in the literature by HCWs during the COVID-19 pandemic (until May 2021), as well as the potential universality of PMIDEs across the globe. These preliminary findings provide further insight into the scope of MI and MD experienced by HCWs and provide information that will be central to further research surrounding the moral experiences faced by HCWs during COVID-19. The authors of this review hope to enhance recognition of the universal challenges HCWs experienced globally during the pandemic, while strongly recognizing the need to dismantle the ‘hero narrative’ toward HCWs, which may perpetuate an unhealthy perception of invulnerable and self-sacrificing individuals (121) and ignore their suffering.

### Study Limitations

It is important to interpret these results with care, given the language constraints of included articles as well as the uneven distribution of articles across geographies, of which a substantial proportion originated from the United States (*n* = 15). Additionally, it is important to recognize the limitations of extracting quantitative and qualitative data from the included articles. Without the original study-level data, it is challenging to ascertain the true relevance of the circumstances associated with PMIDEs. While the extracted quantitative data provided measurable information on the circumstances associated with PMIDEs, it sometimes lacked the detail necessary to appreciate and interpret the specific circumstances. Likewise, accounts extracted from qualitative studies or personal narratives provided the necessary detail but may lack generalizability to the study’s sample as a whole or to the geography of the sample. In order to gain a better understanding of the universality of the PMIDE categories reported in this review, larger studies across the globe are needed, which use mixed method approaches to both identify and quantify the association of certain circumstances to PMIDEs across diverse samples and geographies. Furthermore, the lack of PSP-specific data and literature limits the generalizability of these findings beyond the HCW population. The limited data was partially attributable to differences in defining PSP and HCW, with some articles including paramedics and emergency medical technicians as HCWs and aggregating their results. Future work in this area focused on PSP populations specifically is needed. Further exploration of the impact of cultural and social factors on the experience of MI and MD among HCWs is also necessary to advance understanding of COVID-19 related PMIDEs globally. Finally, it is important to disentangle the longstanding systemic issues experienced by HCWs from specific issues resulting from the COVID-19 pandemic, which will help to further inform discussion and action both during and following the pandemic.

### Emerging Treatments

Despite such limitations, by revealing the scope of MI and MD among HCWs worldwide, these results have important implications for preventative and early intervention efforts aimed at restoring the mental health and wellbeing of HCWs globally. While measures to address MD have primarily focused on the preventative and supportive personal or organizational level efforts (16), insight regarding treatment may be drawn from the field of MI. Emerging approaches for the treatment of MI (e.g., Adaptive Disclosure; Acceptance and Commitment Therapy; Cognitive Processing Therapy) have tended to focus on top-down, cognitively driven approaches (19, 122–124); however, our work focuses on neuroscientifically-guided treatments (125) suggests strongly that approaches that combine top-down, cognitive approaches with bottom-up, physiological and somatosensory-focused approaches, are more likely to achieve success in the prevention and treatment of MI. Accordingly, therapeutic interventions, such as deep brain re-orienting (126, 127) and alpha-rhythm neurofeedback (128, 129), aimed at the integration of somatosensory experience and regulation of visceral response through a combination of bottom-up and top-down mechanisms, are expected to assist in preventative and early intervention efforts for COVID-19-related MI, while also reducing distress driven by lower-level patterns of neural activation.

Notably, regulation of bottom-up driven patterns of response to MI targeted at the midbrain (i.e., the periaqueductal grey) (130, 131), including through breath exercises, may also be expected to further widen the window of tolerance for emotional arousal, thus allowing HCWs to experience a heightened state of regulatory control better situated to the processing of morally injurious experiences. Notably, our own work has revealed that early patterns of emotional abuse (132, 133), and diminished emotional regulation (133), may contribute to the risk for the development of MI. Compassion-focused therapeutic approaches (134, 135) that directly address developmental attachment trauma may further reduce shame and guilt surrounding MI and assist in its processing, particularly when combined with bottom-up, sensory-driven approaches. Finally, given established patterns of perceived social exclusion, poor social support, and a lack of social acknowledgment among HCWs throughout the COVID-19 pandemic, preventative and early intervention efforts focused on the strengthening of interpersonal relationships and enhancing social support would be expected to also assist in addressing MI among HCWs (136), particularly given that meta-analytic research consistently confirms social support as a strong predictor of the development of PTSD following trauma exposure (137, 138).

## Conclusion

On balance, COVID-19 has resulted in novel, potentially morally injurious or distressful, experiences for HCWs across the globe. Although many of these experiences and their associated sequelae appear largely similar across global regions, further research is required to confirm these findings, and identify the prevalence and impact of these experiences within their respective social and cultural contexts. In particular, stigma, discrimination, and violence toward HCWs and their families during COVID-19 may be underreported in some global regions and would benefit greatly from further study and analysis.

## Data Availability Statement

The original contributions presented in this study are included in the article/Supplementary Material, further inquiries can be directed to the corresponding author/s.

## Author Contributions

YX, JL, KR, LB, and MM contributed to the conception and design of the study. YX, JL, KR, and LB contributed to the data collection. YX wrote the initial draft of the manuscript. JL wrote sections of the manuscript. YX, JL, KR, AD’A, RM, AH, RL, and MM contributed to the interpretation of the work. All authors contributed to the critical review and revision of the manuscript.

## Conflict of Interest

The authors declare that the research was conducted in the absence of any commercial or financial relationships that could be construed as a potential conflict of interest.

## Publisher’s Note

All claims expressed in this article are solely those of the authors and do not necessarily represent those of their affiliated organizations, or those of the publisher, the editors and the reviewers. Any product that may be evaluated in this article, or claim that may be made by its manufacturer, is not guaranteed or endorsed by the publisher.
