# Supplementary material for: Potential Circumstances Associated With Moral Injury and Moral Distress in Healthcare Workers and Public Safety Personnel Across the Globe During COVID-19: A Scoping Review
Source: Front Psychiatry. 2022 Jun 13;13:863232. doi: 10.3389/fpsyt.2022.863232 (PMC9234401; doi:10.3389/fpsyt.2022.863232)
Supplement: Supplementary file 2 [file Table_2.DOCX]

# Supplementary Table 2: Geographical distribution of themes associated with PMIDES

| **Risk of contracting or transmitting COVID-19 (n=34)** |
| --- |
| Americas (n=10) (7, 11, 47-54)  Europe (n=8) (55-62)  South-east Asia (n=6) (63-68)  Western Pacific (n=5) (69-73)  Eastern Mediterranean (n=4) (74-77)  Global (n=1) (44) |
| **Inability to work on the frontlines** (n=13) |
| Americas (n=8) (45, 49, 51-53, 77-79)  South-east Asia (n=2) (66, 67)  Africa (n=1) (80)  Europe(n=1) (55)  Western Pacific (n=1) (81) |
| **Provision of suboptimal care (n=33)** |
| Americas (n=15) (7, 11, 48-54, 83-88)  Europe (n=9) (56-58, 60-62, 89-91)  Western Pacific (n=3) (70, 72, 92)  Eastern Mediterranean (n=2) (74, 93)  South-east Asia (n=2) (67, 94)  Africa (n=1) (81)  Global (n=1) (44)  *PPE negatively impacting care (n=8)*  Americas (n=4) (49, 51, 53, 83)  Europe (n=2) (58, 89)  South-east Asia (n=1) (6**7**)  Western Pacific (n=1) (**70**)  *Inability to provide a good death (n=10)*  Americas (n=6) (11, 53, 83, 86-88)  Europe (n=2) (89, 90)  Eastern Mediterranean (n=1) (93)  Western Pacific (n=1) (92)  *Unprepared for novel tasks (n=19)*  Americas (n=6) (7, 48, 49, 51, 54, 83)  Europe (n=8) (57, 58, 60-62, 89-91)  South-east Asia (n=2) (67, 94)  Western Pacific (n=2) (70, 72)  Global (n=1) (44) |
| **Care prioritization and resource allocation decisions (n=12)** |
| Americas (n=6) (11, 48, 51, 83, 85, 86)  Europe (n=2) (89, 91)  Western Pacific (n=2) (72, 92)  Eastern Mediterranean (n=1) (74)  Global (n=1) (44) |
| **Perceived lack of support or unfair treatment by organization (n=16)** |
| Americas (n=8) (7, 47, 48, 51-53, 88, 95)  South-east Asia (n=3) (63, 64, 66)  Europe (n=2) (58, 62)  Eastern Mediterranean (n=2) (75, 96)  Western Pacific (n=1) (69) |
| **Stigma, discrimination, and abuse (n=13)** |
| South-east Asia (n=4) (63, 65, 66, 68)  Eastern Mediterranean (n=3) (93, 97, 98)  Western Pacific (n=2) (82, 99)  Europe (n=2) (58, 62)  Americas (n=1) (47)  Global (n=1) (45) |

Category (n, number of articles)

WHO region of studied population (n, number of articles)
