# Supplementary material for: Potential Circumstances Associated With Moral Injury and Moral Distress in Healthcare Workers and Public Safety Personnel Across the Globe During COVID-19: A Scoping Review
Source: Front Psychiatry. 2022 Jun 13;13:863232. doi: 10.3389/fpsyt.2022.863232 (PMC9234401; doi:10.3389/fpsyt.2022.863232)
Supplement: Supplementary file 3 [file Table_3.DOCX]

**Supplementary Table 3: Moral conflicts associated with PMIDE categories**

| **PMIDE categories** | **Moral conflicts** |
| --- | --- |
| ‘Fear of contracting or transmitting infection’ | Balancing duty to patients and duty to oneself and loved ones |
| ‘Coworkers experiencing a higher risk than oneself’ | Unable to fulfill duty to colleagues due to external constraints |
| ‘Providing suboptimal care’ | Unable to fulfill duty to patients due to external constraints |
| ‘Care prioritization and resource allocation’ | Unable to fulfill duty to patients due to external constraints |
| ‘Perceived lack of support and unfair treatment by organization’ | HCWs risking their lives for an organization who does not protect them |
| ‘Conflict with other HCWs’ | Inappropriate actions of colleagues who should be working towards the same goal |
| ‘Stigma, discrimination, and abuse against HCWs’ | HCWs risking their lives for the public who unfairly treats them |
| ‘Balancing familial responsibilities’ | Balancing duty to patients and duty to family |
