# Supplementary material for: Potential Circumstances Associated With Moral Injury and Moral Distress in Healthcare Workers and Public Safety Personnel Across the Globe During COVID-19: A Scoping Review
Source: Front Psychiatry. 2022 Jun 13;13:863232. doi: 10.3389/fpsyt.2022.863232 (PMC9234401; doi:10.3389/fpsyt.2022.863232)
Supplement: Supplementary file 4 [file Data_Sheet_1.DOCX]

# Supplementary Data 1: Medline search strategy

OVID Medline Epub Ahead of Print, In-Process & Other Non-Indexed Citations, Ovid MEDLINE(R) Daily and Ovid MEDLINE(R) 1946 to Present

1. exp Health Personnel/

2. (healthcare worker* or health care worker*).mp.

3. (healthcare professional* or health care professional*).mp.

4. (healthcare provider* or health care provider*).mp.

5. physician*.mp.

6. surgeon*.mp.

7. nurs*.mp.

8. respiratory therapist*.mp.

9. occupational therapist*.mp.

10. physical therapist*.mp.

11. physician assistant*.mp.

12. psychologist*.mp.

13. exp Social Workers/ or social worker*.mp.

14. exp Emergency Responders/

15. exp Emergency Medical Dispatcher/ or dispatcher*.mp.

16. (police or policing).mp.

17. (firefighter* or fire fighter*).mp.

18. paramedic*.mp.

19. correctional officer*.mp.

20. corrections officer*.mp.

21. law enforcement.mp.

22. or/1-21

23. (moral* not morale).mp.

24. (moral injur* or morally injur*).mp.

25. (morally distress* or moral distress*).mp.

26. Moral integrity.mp.

27. Moral reason*.mp.

28. (moral responsib* or morally responsib*).mp.

29. (ethical responsib* or ethically responsib*).mp.

30. Moral residue*.mp.

31. (moral sensitivit* or morally sensitive).mp.

32. (moral uncertain* or morally uncertain*).mp.

33. (moral ambigu* or morally ambigu*).mp.

34. exp Conflict, Psychological/

35. Moral dilemma*.mp.

36. Ethical dilemma*.mp.

37. (moral conflict* or morally conflict*).mp.

38. (ethical conflict* or ethically conflict*).mp.

39. (moral challenge* or morally challenging or morally demanding).mp.

40. (ethical challenge* or ethically challenging or ethically demanding).mp.

41. shame*.mp.

42. exp Guilt/ or guilt*.mp.

43. exp Betrayal/ or betray*.mp.

44. exp Anger/ or anger*.mp. or angry.mp.

45. or/23-44

46. exp COVID-19/ or covid*.mp.

47. pandemic*.mp. or exp Pandemics/

48. coronavir*.mp.

49. sars-cov-2.mp. or exp SARS-CoV-2/

50. or/46-49

51. 22 and 45 and 50

52. remove duplicates from 51

53. limit 52 to english

54. limit 53 to yr="2020 -Current"

# 
