# Supplementary material for: Potential Circumstances Associated With Moral Injury and Moral Distress in Healthcare Workers and Public Safety Personnel Across the Globe During COVID-19: A Scoping Review
Source: Front Psychiatry. 2022 Jun 13;13:863232. doi: 10.3389/fpsyt.2022.863232 (PMC9234401; doi:10.3389/fpsyt.2022.863232)
Supplement: Supplementary file 5 [file Data_Sheet_2.DOCX]

**Supplementary Data 2: Studies with quantitative measures of moral injury or moral distress**

Out of the 57 articles, two articles used a validated measure of MI and MD respectively to evaluate these constructs. One article measured MI using the Moral Injury Symptom Scale-Health Professional (MISS-HP) (99) while the other article measured MD using the Measure of Moral Distress for Healthcare Professionals (MMD-HP) (60).

A study on 3006 Chinese nurses and physicians found that 41.3% of respondents experienced MI that significantly impaired their functioning (99). Higher MISS-HP scores were very significantly correlated (p<0.001) with higher depression, anxiety, subjective well-being, and all three dimensions of burnout (emotional exhaustion, depersonalization, and greater reduced professional accomplishment) (99). Additionally, HCWs with significant MI demonstrated 4.2 times and 3.8 times greater risk of depressive and anxiety symptoms, respectively (99). Significant protective factors against MI included older age (above 50 years of age), marriage, working as a psychiatrist, and higher educational attainment (MSc) (99). On the other hand, risk factors that demonstrated significant associations include working as a nurse, obstetric gynecologist or pediatrician, female gender, being Buddhist or Taoist, providing direct care to COVID-19 patients, and experiencing violence in the workplace (99).

In a study on 408 Irish HCWs working in pediatric and adult ICUs, MD was reported to be the highest when working with team members who were not sufficiently competent to provide the required patient care (60). Greater MD scores were also significantly associated with the prevalence of PTSD though to a small extent (1.03 OR, CI 1.02-1.06, p=0.0005) (60). Additional factors predictive of higher MD were working in the adult ICU, being redeployed, and exposure to COVID-19 cases (p=0.0001, p=0.0002, and p=0.0001, respectively) (60). Similar to the associations found with MI scores, the difference in MD scores between nursing, medical, and other professions was borderline significant (p=0.05) (60). On the other hand, experience on the ICU and being quarantined were not significant predictors of MD (p=0.08 and p=0.308, respectively) (60).
